# What do we mean by *school entry age*? Conceptual ambiguity and its implications: the example of Indonesia

**DOI:** 10.1080/03050068.2017.1360564

**Published:** 2017-09-08

**Authors:** Bilal Barakat, Stephanie Bengtsson

**Affiliations:** a Vienna Institute of Demography (VID), Austrian Academy of Sciences, Vienna, Austria; b Department of Socioeconomics, Vienna University of Economics and Business, Vienna, Austria; c Wittgenstein Centre for Demography and Global Human Capital (IIASA, VID/ÖAW, WU), Vienna, Austria

**Keywords:** School entry, entry age, Out-Of-School Children, indicators, Indonesia

## Abstract

The age pattern of school entry reflects a complex social and empirical reality that is inadequately captured by a single number. Recognising these complexities in national and international research and policy discourse raises important but neglected questions around the identification of vulnerable groups, the relative value of pre-primary and primary education, as well as the normative powers and responsibilities of governments vis-à-vis parents, and the international educational community vis-à-vis both. This is illustrated by the example of Indonesia, where the official age norm for primary school entry is widely disregarded in practice, with a majority of children starting school one or even two years earlier. Crucially, it is the *compliant* children entering at the statutory age who tend to be from more disadvantaged households, and enjoy no benefit in educational outcomes from their greater maturity.

## 1. Introduction

What do we mean by a country’s ‘school entry age’, when starting school at the official age is actually a minority experience, and what are the potential consequences of such a mismatch between the *de jure* and *de facto* age of school entry? In the case of Indonesia, for example, the official age of school entry is seven years, which is also the figure reported in international databases and used for the purpose of calculating key standard indicators, including the Net Enrolment Rate (NER). However, empirical evidence from numerous data sources shows that the overwhelming majority of Indonesian children have in fact already entered school before their seventh birthday, in many cases one or even two years earlier. This means that any estimates and indicators based on an official entry age of seven fail to capture the actual situation ‘on the ground’.

AIn countries such as Indonesia, where there is a major misalignment between the *de jure* and the *de facto* age of school entry as described, this inability to accurately depict what is actually taking place at the local level can have serious consequences for education research, policy, and planning. Disadvantaged groups may appear privileged, for example, and may be overlooked in national education budgets, or a country’s progress towards universal schooling relative to other countries cannot be accurately gauged. Further, while there is growing recognition globally of the importance of early childhood care and education (ECCE) for learning outcomes in primary and secondary school, and nascent research into the relationship between age, school readiness, and school entry, the capacity of researchers to analyse these issues in a rigorous manner, and of policy-makers to act on this research, are constrained by an ambiguity of who is, in fact, an ‘under-age’, ‘of-age’, or ‘over-age’ student.

Unfortunately, there is no ‘quick fix’ available to clear up this ambiguity and ‘realign’ the *de jure* and *de facto* age of school entry that would enjoy unambiguous legitimacy. However, we would argue that the complex nature and differing rates of child development (particularly in the early years, from 0 to 8), the interrelated factors influencing school readiness, and our still limited understanding of the effect of relative age on a child’s schooling, create an absolute necessity to begin to consider more carefully the concept, definition, and measurement of age at school entry in international education research, policy, and practice. Through a general theoretical analysis of the concept of school entry age and using Indonesia as an illustrative case, this paper aims to open up such a critical discussion about the ambiguities and potential problems resulting from the continued unproblematized use of current school entry age measures.

In Section 2, we approach the issue of age at school entry from various angles, namely in terms of institutional arrangements, measurement challenges, pedagogical, and family perspectives. In Section 3, we continue with a brief overview of primary education in Indonesia that examines its rapid expansion, the official Indonesian policy relating to school entry, and pre-primary education, followed by an empirical analysis of actual entry behaviour and its ramifications for the Indonesian context. Subsequently, in Section 4 we conclude with a consideration of the Indonesian example in light of the general questions previously raised, and conversely, the argument that seeking a general debate on these questions is becoming unavoidable.

## 2. Problematising the notion of a definitive school entry age

It is easy to assume that the school entry age in a given country represents a rule that is well-defined, is perceived as legitimate and mostly respected in practice, and reflects the age considered to be appropriate for following a formal curriculum. Indeed, these assumptions are routinely made, whether explicitly or implicitly, in the compilation of international statistical databases and reports, international and national educational research, and policy debates. However, reality is rather more ambiguous and dynamic. We examine this issue from four different perspectives below, that each focuses on a different essential question. From an institutional/policy perspective, given that schooling is a public institution, who gets to decide which entry timing is legitimate or not, on what basis, and to what extent is it enforced? From a statistical and research perspective, given a complex reality of entry regulations and actual entry behaviour, how do we *define* a single number that is supposed to capture ‘the’ entry age? From a pedagogical perspective, given the significance of the early years as a period of critical (and rapid) child development, what is the ‘right’ age for most children to start school (particularly in terms of more formal learning opportunities), and how do we recognise those who would benefit from an earlier or later start? Finally, from a familial decision-making perspective, given the increasing emphasis on parental choice in relation to schooling, how do parents/guardians determine the ‘right’ entry age *for their own child*, not least in light of decisions made by their peers?

### 2.1. The institutional perspective: the power and legitimacy to set the entry age

At one level, the setting of the official school entry age is a generic example of educational policy-making. As such, questions around the process and legitimacy of setting official age at school entry mirror those concerning issues such as how many years of schooling should be compulsory, at what point formal standardised assessments should be administered, what curriculum should be followed, etc. The answers may differ between settings, with the entry age set nationally, for example, or at the level of province or state, or even left to the discretion of individual schools, within certain bounds. However, of concern is not only: ‘*Who* has the legitimacy power to determine the school starting age?’, but equally: ‘*How* is the decision legitimated, with appeal to what norms?’ While education policy-makers may justify their determinations of the starting age for primary school in a range of different ways, including appealing to the ‘best interests’ of the average child, or to economic competitiveness, or to international ‘standard practice’, often such justifications are not explicitly made, but would need to be inferred.

The reason it becomes particularly important to consider school entry age from an institutional perspective is that the official school entry age is one of the few *policy* variables that directly enter the calculation of international education indicators such as the number or proportion of Out-Of-School Children, indicators that are otherwise based on empirical inputs such as the actual enrolments and corresponding population estimates. In principle, therefore, many countries suffering from significant late entry could reduce the number of Out-Of-School Children ‘at the stroke of a pen’ by formally raising the entry age. While real-life education policy does not, in fact, proceed in such fashion, the point is that it is rather easier to recognise that such a move would lack legitimacy than to define clear criteria for this judgement.

It is a relevant part of the global institutional context that the distribution of school entry ages across countries has steadily concentrated on six years as standard, a threshold that applied in 58% of countries in 1990, 62% in 2000, up to 66% in 2010. Yet it cannot simply be argued that a policy change towards the globally ‘most typical’ entry age is easier to justify than a move away from it. Educational policies that are successful in one country context will not necessarily be equally – or at all – successful in another (Steiner-Khamsi 2012). Indeed, it is easy to find examples where naïve applications of ‘international benchmarking’ in educational policy were obviously inappropriate. The question is not only whether a ‘World Culture’ in education (Carney, Rappleye, and Silova 2012) is in fact emerging, but also whether it is desirable. From a normative perspective, the notion that national education policy must be justified vis-à-vis international standards is certainly not ideologically neutral (Tikly 2016), and may even be interpreted as representing a neo-colonial mind-set (Brock-Utne 2000).

What to make of this objection when – in the Indonesian case, as briefly described above – it is individual parents who appear to be choosing to align themselves with the international standard (of school entry at six years), rather than, or even against, the official government policy (of school entry at seven years)? There are interesting parallels with the question of the language of instruction in the primary cycle: it is not uncommon to see a community preference for teaching in the former colonial language from the start, despite a significant increase in evidence that initial mother tongue instruction is likely beneficial – even for competency in foreign language (including former colonial languages) later in life. The issue is complicated (cf. the range of contributions in Coleman 2011), but it is clear that it is not *necessarily* in the public interest to let parents drive policy by ‘voting with their feet’. This has of course been recognised in industrialised countries also, de-segregation in the USA being an obvious case in point (Baum-Snow and Lutz 2011).

Similarly, the interpretation of national statistics within the framework of international development statistics requires *dialogue* in order to be meaningful. For example, the United Nations’ operational definition of Out-Of-School Children is that of children who are not in school but expected to be according to the school-age range defined by national policy (UIS 2005, 14). However, its agencies recognise that a strictly literal application of the criterion may fail to capture the substantive phenomenon of interest. Home-schooling is a well-known potential source of discrepancy between national legislation (that may allow it, but does not necessarily facilitate the collection of good data on it) and international statistics (that assume that the target value for primary school enrolment and attendance is 100%) (UIS 2005; UNESCO 2015). Elsewhere, where primary schooling is not compulsory (as in Oman, or Nepal, until recently), there is nevertheless a defined ‘school age’ range that serves as the reference for the calculation of international education indicators. In other words, it is decidedly not a prerequisite for national legislation to stipulate a child ‘ought to be’ in school for it to be considered ‘out of school’ for the purposes of international monitoring. Indeed, it is possible for certain individuals (such as pregnant or married girls in Tanzania (Human Rights Watch 2017) and elsewhere (Stacki and Baily 2015)) to be officially *barred* from school, but nonetheless to be considered ‘out of school’ by the international educational development community. All of these examples point to the fact that international education statistics are routinely defined and interpreted with reference not only to national legislation, but also with reference to notions of social equity and justice as framed by international education frameworks.

### 2.2. The conceptual and statistical perspective: summarising varied entry behaviour in a single number

Key education indicators (incl. gross and net intake and enrolment rates, as well as derived measures such as the number of Out-Of-School Children) include the children at the official entrance age to primary education in the denominator. This ‘official entrance age to primary education (years)’ is listed in international statistical databases as a single integer number.

However, even given perfect compliance with the official rules, the single figure is often a simplification. As noted above, the entry age may not be defined at the national level, and may vary across sub-national jurisdictions. Any one of these may be defined with respect to an arbitrary cut-off date that does not necessarily coincide with the beginning of the school year. However, stipulating school entry on 1 September for all children who turned six by 30 June, say, means that strictly speaking the entry age 6.25 (i.e. six years and three months), not 6. If this seems pedantic, note that compared to another country with a 31 December cut-off, this already induces half a year’s difference in the average age of school entrants in the two systems, and that the expected cognitive differences at such a scale are already so large as to motivate the PISA sampling design to define the target population in terms of the month of birth in order to avoid a substantial bias. A rarer complication is that the entry age may consist of a range exceeding 12 months, especially for entry into a flexible reception grade, or if initial entry is permitted at several points during the first academic year.

If already the official rules for school entry can only be approximately represented as a single integer, the correspondence becomes hazier still when dealing with empirical data. The UIS database notes that: ‘The theoretical entrance age to a given programme or level is typically, but not always, the most common entrance age.’ However, no guidance is offered on how to proceed in situations where this is the case, nor is there an in-depth discussion of the ramifications of this limitation. Even for individual researchers who are at liberty and willing to abandon the theoretical figure for the empirical one, there is no established custom of whether ‘the’ actual entry age should be understood to be the mode, median, or mean of the distribution of entry ages, and whether these calculations should be performed on, and the result expressed as, age in whole years or exact age. Nor does there exist a ‘best practice’ consensus on how far the empirical pattern must deviate from the official guidelines in order to be granted precedence in the context of scholarly analysis.

International learning assessments have not settled on a consensus approach either. PISA notably uses the strict age-based definition of the target population already mentioned (albeit under the assumption that this guarantees that test-takers will have completed Grade 7), while TIMSS uses an explicitly hybrid definition taking into account both grade and (average) age. By contrast, both the Early Grade Reading Assessment (EGRA) and Southern and Eastern Africa Consortium for Monitoring Education Quality (SACMEQ) surveys prefer grade-based target populations. This is due at least partly to differences in the groups of countries participating in these surveys, and reflects a reality of relative late entry (both official and de facto) in many low and middle-income settings. This fact is underlined by the fact that the new PISA for Development (PISA-D) initiative, in determining appropriate test content and comparing countries, likewise drops the assumption that participants will have followed a predictable path of entry at a standard age and regular grade progression (Bloem 2013; Carr-Hill 2015) – as indeed it must given that one of its aims is to include children who are not in school.

### 2.3. The pedagogical perspective: the relationship between age, early learning, and ‘school readiness’

Empirical research on the effect of entry age on school outcomes can look back on a history that extends at least a hundred years (Winch 1911). However, as interest in ECCE increases in the international community, researchers, and policy-makers in some countries (particularly the high income group) have re-visited the notion of ‘school readiness’, and whether entry to Grade 1 should be decided based on age and/or an assessment of a child’s development.

Horstschräer and Muehler’s (2014) research on school readiness describes the process in Germany by which developmental school entrance screenings are factored in alongside a child’s age to determine the best time for a child to enrol in Grade 1. Children who are recommended to start school are more frequently girls, are older, had a higher birth weight, and longer experience in child care. Further, children who are above official school entry age (six years, in the German case) are always recommended, while children with a low social status tend to be recommended less often. This study lends credence to the idea that a child’s age should be more of an indicative than a normative guideline when considering whether or not that child is ready to begin school, though, as has been previously mentioned, this is not how age at school entry is treated for the purposes of national and international statistics.

**Figure 1.**
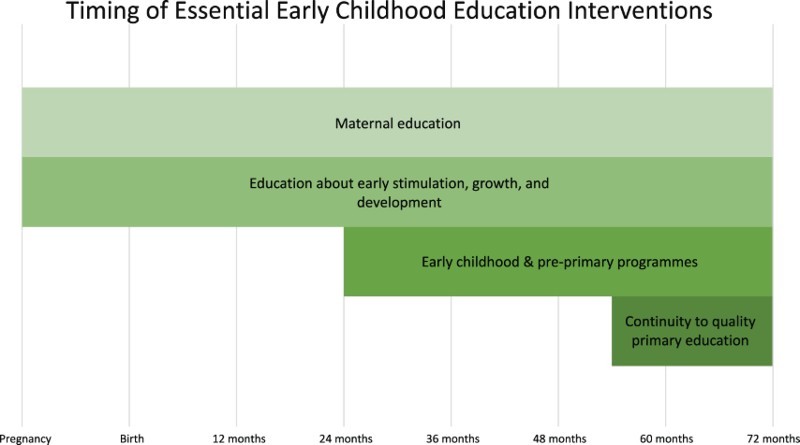
Recommended timing of Early Childhood Development interventions. Adapted from Denboba et al. (2014).

Figure 1 illustrates the recommended timings emerging from the research of essential Early Childhood Development (ECD) interventions in the education sector, according to the age of the child (from conception through to 6 years/72 months). General education of mothers and specific education on healthy child development for caregivers are two interventions spanning the entire time period, and will have important implications for the stimulation and learning of a child from birth. More formalised ECCE and pre-primary programmes are highlighted as key interventions starting from the age of about 2 years (24 months). Finally, ensuring continuity between ECCE and pre-primary programmes and quality primary education should be a priority concern starting from the age of approximately four and a half years (54 months) upwards.

Two relevant observations may be made here: (1) that there is an underlying assumption among early education experts that children are likely to start formal primary education at age six (reflecting the global norm), and (2) that a conscious effort is required in the two years leading up to a child’s sixth birthday to help that child prepare for primary school in order to compensate for differences in readiness and preparedness for school.

While it is clearly true that such differences exist between individuals and families, it is less clear why general differences would exist between countries in terms of the age at which its children typically reach a given level of maturity. However, the question is: ready *for what*? The effective demands that different national school systems place on primary entrants do indeed differ. This concerns both the complexity of the script and alphabet to be taught, for example, but also the typical distance and transport modality of the school commute. While we are not aware of any systematic analysis of the extent to which the variation in entry age across countries correlates with such factors, it is clear that in principle there may well be pedagogically reasonable considerations that would lead to adoption of different entry norms.

In order to respect individual developmental differences, an alternative to relaxing the entry age threshold is to relax the grouping into age cohorts inside schools, opting, for example, for a multigrade approach. In fact, emerging research on the Escuela Nueva programme in Colombia suggests that multigrade teaching may be preferable to monograde teaching, because it requires greater flexibility and thus allows for more opportunities for both cooperative and more individualised learning (Luschei and Zubaidah 2012). However, it is still more commonplace to consider multigrade teaching to be a major challenge to positive learning outcomes, particularly in contexts where multigraded classes have arisen out of necessity rather than by design, such that millions of learners around the world are taught by ‘invisible’ multigrade teachers attempting to teach a curriculum designed for monograded classrooms (Little 2007). In resource-poor contexts in particular, where teachers are more likely to miss out on adequate professional development opportunities to learn how to respond to a diverse range of learning needs, it is clear that a limited understanding of school entry age serves to further muddy the waters of educational planning to improve learning outcomes for all.

### 2.4. The family perspective: absolute age, relative age, and getting ahead

A large body of research literature grapples with the ‘age effect’ on the academic (Sharp 1995; Sprietsma 2010; Peña 2017; and many more) and athletic success (Musch and Grondin 2001; Schorer, Wattie, and Baker 2013), as well as psychological well-being (Thompson, Barnsley, and Battle 2004; Matsubayashi and Ueda 2015) of children. There is some evidence that older children may outperform their younger peers, but the universality or contingency, magnitude, and persistence of this effect are subject to debate. The nuance of the research has not stopped the popularisation of selected findings from providing a motivation for some families to exploit any room for manoeuvre with respect to school entry to maximise their child’s (perceived) chances of educational success.

In fact, there is not one but two ‘age effects’ to consider. The *absolute age* at which certain cognitive tasks are encountered deals with questions such as whether children who learn to read at ages six or seven eventually catch up with children who learned to read at age five, say. By contrast, the question concerning *relative age* is whether being younger or older within their peer group creates an initial (dis)advantage in physical and cognitive maturity that leads into a self-reinforcing feedback loop of lowered or heightened expectations and self-confidence.

To the extent that there is a benefit to being relatively older, the relationship with age is certainly non-linear. While those students who are among the older *within the appropriate age range*, that is, older at the scale of months, are generally found to enjoy an advantage over their younger peers (e.g. Horstschräer and Muehler 2014), this turns into a disadvantage for students who are ‘over-age’, that is, older than their peers at the scale of years (Jeronimus et al. (2015) observe a corresponding inversion of the relative age effect for those who repeated a grade).

Under a strict entry regime, absolute, and relative age cannot be easily disentangled, since the only variation in age in a given grade is within classrooms. According to the current state of knowledge, there appears to be nothing *inherently* better or worse about starting school at age six or seven, and the exact same age-grade combination may be ‘overage’ in one context but not in another. Accordingly, most studies focus on ‘relative age’ and typically attempt to explain its effects in terms of the actual age (Sykes, Bell, and Rodeiro 2009) of children’s peers. Confusingly, some research designs seek to *exploit* the occurrence of deliberately deferred entry, while others are premised on *ignoring* it. The statistical argument in favour of analysing the theoretical relative age determined by the official admissions rule rather than the actual empirical relative age of students (Sprietsma 2010) is to avoid potential distortion through endogenous selection. Such selection occurs if the characteristics of parents who deliberately enter their children earlier or later than the standard age are correlated with the educational or other outcomes that are being investigated. However, this methodological rationale relies on the crucial assumption that such deviations occur at the margins, that ‘assigned relative age must be correlated with observed relative age’ (Bedard and Dhuey 2006, 7). Unfortunately, the claim that ‘since most students enter school on time and are never retained this is easily satisfied’ (Bedard and Dhuey 2006) may well be the case for their specific study but unfortunately cannot be assumed in general (and manifestly fails in the Indonesian context). The statistical reasoning breaks down and cannot be salvaged when actual entry behaviour ceases to bear any resemblance to the official rules.

As a matter of fact, deferred entry creates poorly-understood conceptual and methodological challenges long before a majority of families engage in it. It is mathematically trivial to specify plausible age patterns of entry such that, with a September school start, for example, children with birthdays in December are *more* likely to be among the oldest 40% in their class, but *less* likely to be among the oldest 30%, than children with birthdays in August, if some of the latter (but no-one else) defer entry for a year. Understanding the effect of relative age therefore requires a much more careful conceptual justification of who *exactly* are considered to be ‘the older children in the peer group’ than researchers have engaged in until now.

Families strategizing around the timing of school entry face competing goals of ‘getting a head start’ for their child by beginning exposure to the school curriculum at an (absolute) age as early as possible, but at the same time of attempting to place them among relatively younger peers. This tension may partly explain the emergence of a relatively stable ‘equilibrium’ entry into primary school around six years of age, and the extension of formal education into early life in form of a non-competitive pre-primary phase rather than a race to ever-earlier entry into primary school proper.

As the overview above shows, it is very much contingent on perspective and purpose, not only what the age of school entry should be, but even what it actually *is.* The risk of ignoring this ambiguity, and of international agencies and researchers taking the *de jure* definition at face value as a close approximation to manifest *de facto* entry behaviour, is illustrated by the example of Indonesia, which we discuss in some detail below. There, the gap is both large and largely unrecognised, and, as a result, obscures the fact that formally ‘on-time’ students may actually be on their way to becoming a relatively disadvantaged population deserving of concern. Our case study of Indonesia opens with a brief introduction to primary and pre-primary education in Indonesia, before we present the three key quantitative patterns that emerge with regards to school entry age, and conclude with the implications that these patterns may have on Indonesian children’s learning opportunities.

## 3. An empirical case study: Indonesia

### 3.1. Primary and pre-primary education in Indonesia

Indonesia has one of the largest formal school systems worldwide, with around 30 and 20 million children enrolled in primary and secondary education respectively, making it third in size only behind China’s and India’s. This scale results from a large underlying population size in combination with relatively high rates of educational participation. Given the scale of the education system, the large gap between the *de jure* and the *de facto* age at school entry, once recognised, highlights the urgency in making progress towards an integrated approach to the general issues outlined in the previous section.

Beginning in 1973 with a policy directive to build a primary school in every village, Indonesia’s government has taken a number of steps to achieve the expansion of educational participation, including making six and then nine years of education compulsory (in 1984 and 1994 respectively), and then implementing a range of decentralisation policies, pro-poor initiatives, and teacher quality improvement programmes (Suharti 2013). As a result, the gross enrolment ratio (GER) in primary school rose from 79% to 119% between 1974 and 1984 and has remained above 100% in the past two decades (ibid.). In 2005, the Indonesian government put into place legislation that required all primary school teachers to have a four-year post-secondary degree by 2015 (Luschei and Zubaidah 2012), which may have helped to reassure parents that quality of education would improve alongside access to schooling.^1^

In 2010, a GER of 112% stood alongside a *net* enrolment ratio (NER) of 95%. By construction, discrepancies between the GER and NER are due to the enrolment of children outside the official school age bracket. But unlike the comparatively more typical concern with *overage* enrolment, in Indonesia the size of the gap between the GER and the NER in Indonesia can be explained by the large number of officially *underage* children currently enrolled in primary school – close to half of students enrolled in Grade 1 (including Islamic primary schools) were underage in 2010 (Suharti 2013).

The 2003 Indonesian Education Act protects the right to receive basic education for ‘[e]very seven to fifteen years old citizen’ (Article 6, 1), serving as the basis for the official interpretation stipulating an entry age of seven. However, this statement is not explicitly qualified to imply that actual entry is deferred until the beginning of the next school year (when the child is actually aged seven). If this Article is interpreted as conferring an immediate right, it is one that can only be satisfied either by allowing the uncommon practice of entry throughout the school year (to coincide with each child’s seventh birthday), or by enrolling children at the beginning of the school year during which they will turn seven. The latter option would of course simply amount to an effective entry age of six. Indeed, the Act also states that ‘[e]very citizen can enrol in a compulsory basic education programme at the age of six’ (Article 34, 1). In other words, present legislation arguably at least allows for an alternative reading in support of a regular entry age of six years, and a large proportion of families does in fact avail itself of this opportunity.

As we will argue later in this paper, learning inequalities begin early and intensify over time (Rose and Alcott 2015), therefore having a more accurate picture of what happens at the start of primary school (and indeed, a more complete profile of Grade 1 entrants) is crucial to understanding and addressing the sources and consequences of inequalities both in learning and attainment, inequalities that remain substantial. As a result of school expansion, the average number of years of schooling rose from around 6 to almost 8 years among members of the Indonesian population aged 15 years and over between 1993 and 2010 (Jones and Pratomo 2015), but this increase was uneven. While a gender gap of 0.8 years in favour of males remained, it is now far exceeded by spatial and socio-economic inequalities. These inequalities continue to present reasons for concern. Those in urban areas experience on average 2.7 years more schooling than their rural compatriots (Suharti 2013), for example. Further, there are concerns about the quality of education in rural versus urban areas – teaching of heterogeneous classes may present a challenge for teachers in remote, rural areas, and teacher absenteeism is nearly twice as high in multigrade schools than non-multigrade schools (Luschei and Zubaidah 2012).

Indonesia has, since the early 2000s, begun placing some priority on the ECD sector, not least by including ECD in the 2003 Education Act, and launching its first-ever ECD Census in 2011 (Denboba, Hasan, and Wodon 2015). This development is reflected in increasing uptake. The GER and NER for pre-primary education in Indonesia were 58.16% and 40.45% respectively in 2014, which is a significant increase from 2006 (34.6% and 24.71% respectively). Further, the data suggest in 2014, the number of new entrants to Grade 1 with early childhood experience was 61.31%, up from 37.22% in 2004 (but down from 70.30% in 2012).

However, while ECCE provision is clearly increasing for children aged four to six (including pre-primary education for five- to six-year-olds), ECCE is not compulsory and levels of public spending on this sector are extremely low (Al-Samarrai and Cerdan-Infantes 2013). Indonesia tends to perform less well than other countries in three areas where the World Bank’s SABER analytical tool for ECD^2^ has been applied, including programme coverage, equity, and compliance with standards across the range of ECD services (Denboba, Hasan, and Wodon 2015). The SABER study for Indonesia highlighted low coverage of pre-primary education enrolment as a particular problem.

While the majority of new Grade 1 students have some early childhood experience, there is a significant proportion of students who do not, potentially putting them at a disadvantage to their peers. In fact, high returns to early childhood education have been found both in Indonesia and globally (Al-Samarrai and Cerdan-Infantes 2013). A recent Early Grades Reading Assessment (EGRA) conducted with Grade 2 students in Indonesia found that the largest non-regional difference in oral reading fluency across all demographic variables came from pre-school attendance (Stern and Nordstrum 2014). Jones and Pratomo (2015) have demonstrated that current early childhood provision in Indonesia reinforces the advantage of the better-off, with only 36% of four to six-year-olds from the poorest quintile of families attending ECCE programmes in 2010, compared with 68% from the richest quintile.

Following this brief discussion around the provision of primary and pre-primary education in Indonesia, we will now present our analyses of quantitative data on school entry age in Indonesia.

### 3.2. Quantitative data sources

Multiple data sources are analysed here, for two reasons. Firstly to validate them against each other, and secondly because specific variables are available in some surveys, but not others. This includes the *reason* for non-entry, for example.

We focus on the three Indonesian *Demographic and Health Surveys* (DHS) from 2003, 2007, and 2012. An additional source of relevant data is provided by the *SUPAS* intercensal survey that has been conducted regularly every five years for several decades and therefore provides a useful perspective on changes over time. The *SUSENAS* (Statistics Indonesia (BPS) 2012) is another large-scale multi-purpose survey that has been collected by the national statistical office of Indonesia with increasing frequency (currently quarterly, with a rotating panel). The *Indonesia Family Life Survey* (IFLS) (Strauss et al. 2009; RAND Corporation 2014) is a longitudinal household survey representative of 13 Indonesian provinces containing about 83% of the national population. It is included in the analysis because it contains exact birth dates for all children, as well as reported entry ages.

### 3.3. Quantitative patterns

The analyses are grouped into three broad areas. The first directly examines the distribution of the age of school entry, as well as the question of when and where the present patterns emerged. The second relates the responses of parents to the observed entry age in order to shine some light on their understanding of the meaning and importance of the official criterion. The third compares entrants aged six and seven, examining whether the former should be considered ‘early’ entrants or the latter ‘late’, in terms of the socio-economic determinants of their status and of their schooling outcomes.

Multiple data sources are analysed here, for two reasons. The first is to validate them against each other, and because specific variables are available in some surveys, but not others. This includes the

#### 3.3.1. Manifest entry behaviour over time and space

To begin with, we can consider simply the percentage of five, six, and seven-year-olds attending school according to different recent surveys. These are displayed in Table 1. For sources that only record ages in whole years, the timing of the survey is indicated and should be considered in relation to the beginning of the school year. In those cases, attendance ratios for those aged 6 at the beginning of the school year are estimated by regressing on the interview dates (Barakat 2016).

**Table 1.** Age-specific (ever) attendance ratios of children aged five to seven at time of survey (‘nominal’) and aged six at beginning of school year, either known (if data contains birth months) or estimated (starred).

Source	Year	Fieldwork	6 at entry	Nominal 5	Nominal 6	Nominal 7
DHS	2003	Oct 2002 to Apr 2003	–	8	56	91
SUPAS	2005	June 2005	88	7	44	86
DHS	2007	June 2007 to Feb 2008	–	22	74	95
IFLS	2007	Nov 2007 to Apr 2008	84	5	51	93
DHS	2012	May to July 2012	84*	4	37	89
SUSENAS	2012	Mar, Sept 2012	87*	7	57	96

In sum, at least five independent data sources all agree that a substantial majority of children in Indonesia are already in school by the time they turn seven years old. While age misreporting can in general be a concern with household surveys, the reported age pattern shows no evidence of misreporting that would inflate the apparent enrolment of six-year-olds. Moreover, the results are robust to including only children whose parents could produce the child’s birth certificate.

**Figure 2.**
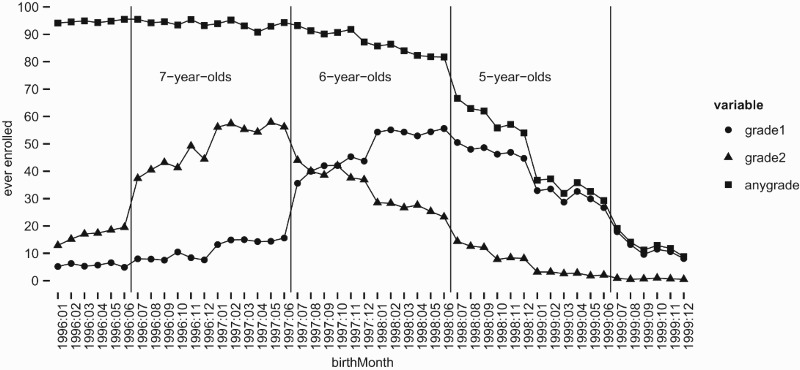
Share ever enrolled in school year starting July 2004, by birth month. Data: SUPAS.

The availability of more detailed birthdate data in some surveys allows for an analysis of entry behaviour by age in months. Both SUPAS and IFLS contain the necessary information, and give similar results, but SUPAS is preferred for this particular analysis because of the larger sample size. The data were collected in June 2005. Prospective entrants (to enter school after the summer) are explicitly specified in the questionnaire to be recorded as ‘not/not yet attending’. Accordingly, the attendance status is defined strictly with reference to the school year 2004–2005, and the ‘age at entry’ is relative to July 2004. The results are displayed in Figure 2.

As above, age six is the entry age reported for an absolute majority of children. This is true independently of the month of birth, and in particular even for those who had turned six only recently at the time school starts. Evidently, 90+% of ‘almost seven-year-olds’ (red) attended, 80–90% of the ‘only just six-year-olds’ (blue), and 50–70% of those turning six by the end of the calendar year (green). The discontinuities occur exactly at the psychological thresholds of being nominally five or six when school started, and turning six before or after the new calendar year, further bolstering the credibility of the age data.

By necessity, these figures are based on the subsample of children with known birth month, which is likely to be biased away from disadvantaged households. This explains why the attendance of six-year-olds is at the upper end of the range if we compare different surveys. However, the proportion of missing birth month data is too small to affect the overall conclusion.

The results on entry by years of age are further corroborated by the reports on the 2012 PISA study. In the PISA sample, too, age six is the modal entry age, and less than 40% of students started school at age seven or older (OECD 2013, Figure IV.2.2.). While this latter figure is slightly higher than those based on the other sources, the sample is smaller, based only on children still in school, and relates to entry at an earlier point in time than most of the other surveys.

Different waves of SUPAS show why this issue has not been widely recognised to date. As recently as the mid-1990s, the *de jure* and *de facto* entry ages did in fact match. There had always been a considerable amount of early entry, and a continuous decline in entry at ages seven and above. Indeed, this had been noted with respect to data as early as 1997 (UNESCO Division of Statistics 1997, 10). However, the final move to mass entry strictly before seven only occurred around the turn of the millennium.

**Figure 3.**
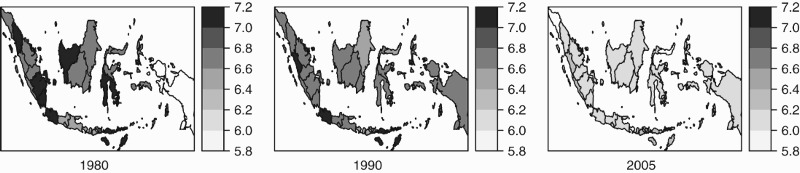
Spatial distribution of average age of entry of first-graders, in months. Data: Censuses 1980, 1990, SUPAS intercensal survey 2005.

Indonesia is a large country, with a sizeable population dispersed over a wide geographical area. In order to verify the validity of generalising the findings above at the national level, the following maps (Figure 3) display school entry dynamics at the provincial level. Evidently, the trend towards earlier entry has been universal, with no obvious spatial concentration. In particular, it is certainly not merely a ‘(Greater) Jakarta effect’.

#### 3.3.2. Parental perceptions and age reporting

It is instructive to go beyond the manifest entry behaviour and examine *perceptions* of the appropriate for school entry. Both SUSENAS and the IFLS query the *reason* for non-attendance for children not in school. In addition, the IFLS includes the *reported* entry age, providing indirect evidence on how the age threshold criterion for school entry is interpreted by parents.

Turning first to the explicit reasons for non-attendance, displayed in Table 2, both sources show that, in absolute terms, only a small minority refrains from enrolling their six-year-olds in school because of considering them ‘too young’. The figures for IFLS from July 2007 and from SUSENAS for Sep 2012 are actually highly consistent, taking into account that the latter includes up to two additional birth months of children who only just turned six. The distinction between those who turned six or seven in the calendar year 2007 is worthwhile. Even among those six-year-olds who did not enter school in July 2007, close to a fifth did so for stated reasons other than age; Among those who turned seven by the end of the year, this share rises to almost a third. More recently, the seven-year-olds in the 2012 SUSENAS sample similarly comprise largely of children who were six at the beginning of the school year; Nonetheless, not one (!) of them was reported not to have entered yet on account of being ‘too young’.

**Table 2.** Non-entry reportedly due to age.

Survey	Age	Sample size	a. Not attending (%)	b. Not attending because ‘too young’ (%)	b/a
IFLS	6 in Jul ’07	866	15	12	.82
SUSENAS	6 in Sep ’12	5591	29	26	.90
IFLS	6.5 in Jul ’07	448	9	6	.68
SUSENAS	7 in Mar ’12	5991	5	0	.00

**Figure 4.**
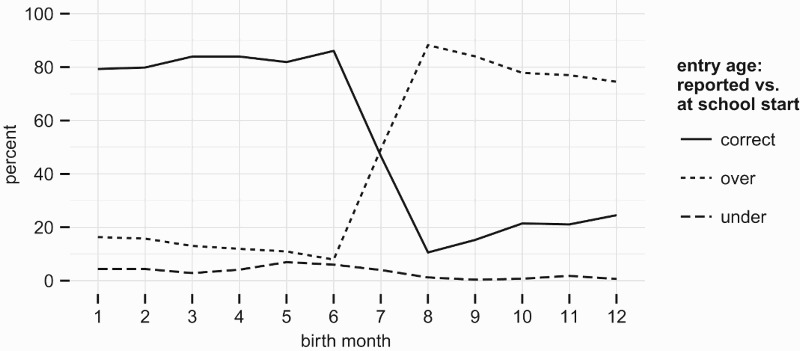
Parental reports of entry age vs. actual age at school start. Data: IFLS 2007.

**Figure 5.**
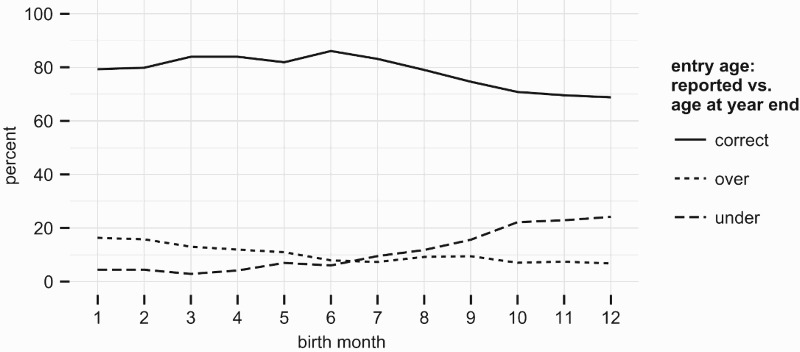
Parental reports of entry age vs. actual age at end of calendar year of entry. Data: IFLS 2007.

An additional value of the IFLS data is the age at school entry is observed both implicitly, given exact birthdates and enrolment status, and explicitly as an additional question in the survey. Figures 4 and 5 compare how the reported entry age relates to the actual age of the child at the beginning of the school year on the one hand, and the child’s age by the end of the relevant *calendar* year on the other.

Children whose birthday falls between the beginning of the school year and the end of the calendar year are very likely to have their entry age ‘overstated’ relative to their age at the beginning of school. A parsimonious characterisation of this response behaviour is that most families interpret the entry age threshold in terms of the age reached in the *calendar* year of school entry, and only children born very early/late in the year are likely to be recognised as being over or under-age.

#### 3.3.3. Differential schooling determinants and outcomes by entry age

**Figure 6.**
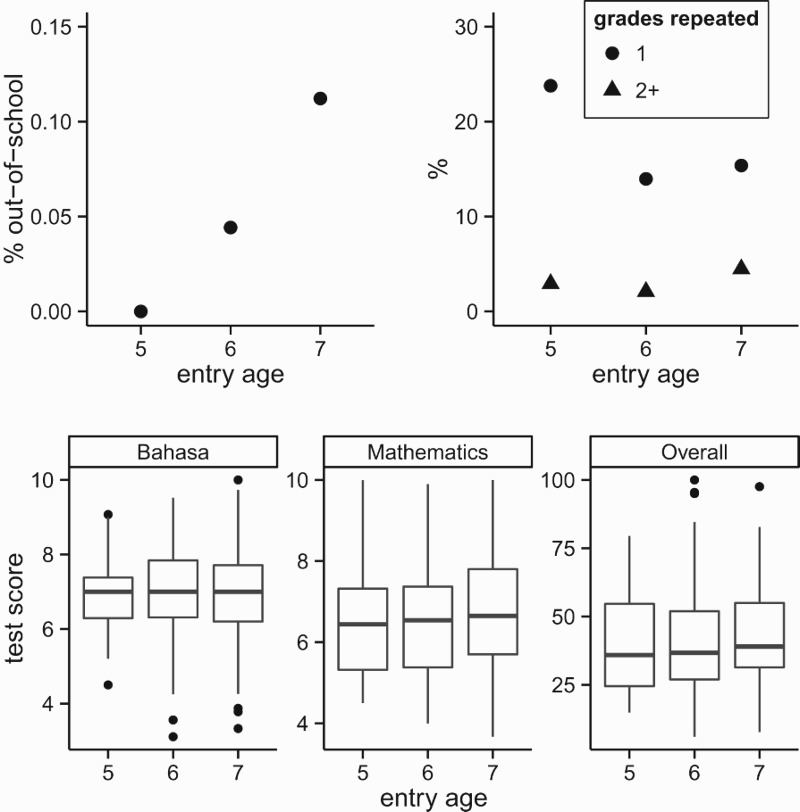
Schooling outcomes among 14-year-olds. Data: IFLS 2007.

Key schooling indicators, namely attainment, repetition, and learning assessments, for children aged fourteen when observed in the 2007 IFLS, conditional on their age of school entry, are shown in Figure 6. Consistently, these results show no significant differences by entry age, or that – if anything – those entering at age six experience better outcomes than those entering at the official entry age of seven. This conclusion also holds when examining the regression coefficients of entry age indicator variables, with sex, urban/rural residence, and education of the household head as additional controls.

The above results suggests that while Indonesia does score rather low in PISA, this cannot be blamed on widespread ‘premature’ entry. In fact, research on Early Childhood Education shows that if there is an area where Indonesian kindergarteners are behind in international comparison, it is in reading (World Bank. Indonesia Office 2010), but the PISA reading score is actually the one that improved (!) the most between 2003 and 2012, exactly when the corresponding entry cohorts became even younger.

As it were, it is the ‘compliant’ children who enter at age seven that tend to come from socioeconomically somewhat more disadvantaged backgrounds, not the ‘irregular’ entrants at age six (Table 3). In particular, the former report a lower education of household head, lower ownership of the family dwelling, and are less likely to have electricity or sanitary facilities at home. As a result, failing to acknowledge them as ‘de facto *late* entrants’ is to actively increase their marginalisation and amounts to ‘anti-poor’ policy.

**Table 3.** Regression coefficients of the probability of school children who were aged seven at the beginning of the academic year attending Grade 1 (i.e. entry at age seven) as opposed to Grade 2 (i.e. entry at age six).

	Estimate	Std. error	*z* Value	Pr(>|*z*|)
Dwelling not owned	0.31	0.14	2.2	2.9e−02
No toilet	0.35	0.12	2.9	3.6e−03
No electricity	0.41	0.15	2.7	6.7e−03
Head’s education at most primary	0.68	0.14	4.7	2.3e−06

Notes: Only regressors significantly different from zero at 0.95 confidence are shown. Other variables are: rural residence, lack of TV, gender.

### 3.4. Implications

While these results together suggest a deliberate change in entry behaviour, a conclusive explanation for its motivation remains elusive. There are, however, a number of contextual factors that may point in the direction of plausible explanations.

As noted above, the timing of accelerated early entry aligns with the Asian financial crisis. While negative effects on education were remarkably small. Nevertheless, the crisis did delay further educational *expansion*, while incidentally the primary school-age dipped during the mid-1990s to mid-2000s. The early part of this development was already credited with contributing to increased early entry (Oey-Gardiner 2000), and the implied relaxed capacity constraints in primary schooling created a conducive environment for the acceleration of that trend. In addition to this supply-side dynamic, factors on the demand side were likewise favourable. Educational resilience in the face of the crisis resulted from provoking families’ deliberate and determined commitment, who largely protected their children's educational opportunities at the cost of other sacrifices (Cameron 2001; Frankenberg and Thomas 2017). Indeed, there was a marked increase in *pre*-primary enrolment around the same time. Moreover, as part of a longer trend, the labour market returns to education generally declined during that period, with a much larger premium for secondary schooling than primary schooling (Purnastuti, Miller, and Salim 2013). In effect, the amount of schooling typically required to earn a living increased, and in order not to delay their children’s entry into gainful work, even poorer families faced an incentive for school entry earlier than the official age.

When we consider the quantitative patterns of changing school entry behaviour in relation to the recent Indonesian EGRA study discussed earlier, which found significant positive differences in oral reading fluency for being ‘of-age’ (Stern and Nordstrum 2014), we begin to understand what is at stake when it comes to conceptual nebulousness of school entry age. The survey targeted Grade 2 students and was conducted in March and April, that is, late in the school year, which starts in July. Nevertheless, ‘of-age’ for purposes of the study was taken to mean seven *or* eight years of age, with no distinction drawn between students who had just turned seven, those who were seven turning eight, and those who had turned eight towards the beginning of the academic year. While it is not uncommon to allow for a buffer of one year above the statutory age corresponding to a given grade before considering a student ‘over-age’, the above constellation means that effectively the bracket was extended downwards instead of upwards. As noted, the EGRA operationalisation of ‘of-age’ included seven-year-olds in Grade 2, who must therefore have enrolled in Grade 1 at an age strictly below seven, and who were ‘under-age’ according to official school policy. Conversely, some students who entered school at the official age of seven, and turned eight during the course of Grade 1 and turned nine during the course of Grade 2 were effectively classified as ‘over-age’. This provides a salient example of the ambiguity arising from a large difference between the official age threshold, and the actual distribution of student ages found in classrooms, which must be the overriding consideration for studies that aim to investigate the effect of being older or younger than your peers.

Results from the Indonesian EGRA show that in addition to students with early childhood experience and of-age students doing better on the reading assessments, girls tended to outperform boys, and students from better-off communities tended to perform better than those from poorer, more remote communities (Stern and Nordstrum 2014). Notably, these characteristics align closely with the selection of students who would likely have received a recommendation for relatively earlier school entry under a formal assessment of school readiness such as the one in Germany.

Further, the generally positive impact on primary school learning outcomes of ECCE exposure in general may mask a negative impact of low quality programmes. Indeed, while the government has succeeded in establishing several important ECD delivery mechanisms and infrastructure standards, many ECCE teachers are not qualified and only a small number of ECCE centres are actually accredited (Denboba, Hasan, and Wodon 2015).

One of our central arguments is that *the equity implications of socioeconomic participation patterns can only be assessed if the age patterns of participation are taken into account*. Alatas et al. (2013) note that while different types of ECCE services are intended to cater to specific age groups, these age groupings are not consistently adhered to in practice: Local conditions, service availability and family preferences mean that some four to five-year-olds may still be attending playgroup, rather than kindergarten, and some six-year-olds may already have started primary school. The present analysis provides comprehensive empirical backing for the contention that the age spread is even larger than thought, and interacts with socio-economic status.

Given the questions surrounding the quality of ECCE provision, a six-year-old in ECCE may receive less benefit than a peer in primary school, where there are more trained teachers, and more institutionalised standards of quality. If ECCE participation and early primary school entry were in competition, this would tend to reduce inequality, as a seven-year-old enrolled in Grade 2 with no ECCE experience may well be at an advantage in terms of positive child development and learning outcomes compared to a seven-year-old enrolled in Grade 1 with one or even two years of poor quality ECCE experience. However, our findings show that, in fact, the nominal socio-economic differences in ECCE participation *understate* the underlying inequality: four-to-six-year-old children of more advantaged groups are more likely to attend ECCE – even though they are also more likely to start primary school earlier. In other words, the difference in the overall duration of education is even greater than it seems.

## 4. Discussion

To summarise the empirical school entry behaviour in Indonesia, multiple independent data sources conclusively demonstrate that the current official rule for school entry in Indonesia appears to exert little normative pressure and has in fact been consistently disregarded for some time now, and throughout the country. A simple re-interpretation of existing rules in terms of prescribing entry for those turning seven by the end of the *calendar* year would align with the popular interpretation, and go part way to legitimising the entry of some six-year-olds (at the beginning of the school year) as being ‘on time’; However, entry rates are so high among those ‘only just six’, and even among five-year-olds, that this would only partly resolve the issue, and formally ‘early’ entry would still be the majority experience. Standard research designs to study the effects of relative entry age, as well as customary interpretations of international education statistics, either implicitly or explicitly rely on the statutory entry age actually representing the majority experience, and are thrown off by the entry pattern just described.

Does this state of affairs amount to a case for changing the official entry age to align with actual practice? And do our deliberations on this question with respect to the specific example of Indonesia lead to a re-assessment of the general issues outlined in Section 2?

As discussed there, alignment with popular demand and international standard practice is not by itself a sufficient justification for a policy change. As demonstrated in our analysis, there are, however, other reasons why such a move could be justified: (a) the average entry age is *so* far below the statutory threshold of seven, that it is lower than the average entry age in some countries with entry at age six, but a high prevalence of voluntary deferred entry, such as Germany; (b) this state of affairs is not a glitch or fad, but a long-term trend has been going on for at least 15 years – in other words, for an entire school generation; (c) finally, this is not merely manifest behaviour, but in addition there is little effort by parents to pretend their children entered at age seven when they did not, pointing to a weak normative force of the official entry age. However, while these facts strengthen the case for a policy change, they may not be sufficient.

Moreover, whether the official entry age should be changed to reflect actual behaviour is actually too limited a question. Apart from the fact that such a policy change would take time to enact, it could only affect the future. The extent to which the *past and present data* should be re-interpreted by national and international agencies and policy researchers is therefore a separate, and more fundamental question, namely: what is the ‘deep’ definition of *Out-Of-School Children*, *late entrants*, *overage enrolment* and other indicators of educational disadvantage and exclusion?

This way of posing the question draws attention to the fact that the answer is quite clear. Serving as tools to identify inequity is the *raison d'être* of these indicators, and the definition is most appropriate that best serves this purpose. Arguably then, it is the findings regarding the differentials in socio-economic background and schooling outcomes that are crucial to the interpretation of the prevalence of entry at age six. To recap, entry at age six is not associated with worse outcomes, but entry at age seven *is* associated with general socio-economic disadvantage. It is therefore socially regressive to treat those entering at age six as ‘early/premature entrants’, instead of treating those entering at age seven as ‘late entrants’ and deserving of special concern. This concern should trump a strictly legalist reading of the data. Certainly for independent researchers, analysts, and advocates, and perhaps even for international agencies and organisations. Those among the former who are interested in studying the effects of relative age have no choice but to account for the fact that the official entry age is disregarded in practice, regardless of whether this is acknowledged by the national administration. Those among the former who study general socio-economic differentials need to do so in order to avoid missing inequities that are otherwise obscured. The latter, finally, face a balancing act between the need for relevance and comparability in their indicators and the demands of consistency with the national administrative perspective on the other.

While these issues are brought into stark relief by the example of Indonesia, they are by no means limited to it, or to developing countries. For example, arrangements in England possess significant potential for confusion as to the effective school entry age (Sellgren 2015), and in parts of Germany, a direct age-grade correspondence is loosened as a matter of policy through a flexible entry phase (Faust 2006) based on an entry age bracket rather than simple threshold. It is only a matter of time until there will be no choice but to adapt some of our statistical and conceptual tools of educational research to such definitional ambiguities. With regard to statistical measurement, renewed consideration might be given to global indicators defined in terms of a universal age bracket well clear of entry ambiguity, such as enrolment among those aged 8–12, instead of enrolment of the nationally-defined ‘school-age’ population. The task of coming to conceptual terms with the fact that ‘the school entry age’ is not a single, well-defined number, is likely to be the greater challenge.

## Notes

1 It should be noted, however, that according to Luschei and Zubaidah (2012), in 2006, only 17% of practising teachers had the appropriate degree and more than 1 million would need to have their qualifications upgraded.

2 The Systems Approach for Better Education Results (SABER) is a World Bank initiative which evaluates the quality of education policies against evidence-based global standards to produce comparative data and knowledge on education policies and institutions.
